# Magnetic Particles for CTC Enrichment

**DOI:** 10.3390/cancers12123525

**Published:** 2020-11-26

**Authors:** Peng Liu, Pascal Jonkheijm, Leon W. M. M. Terstappen, Michiel Stevens

**Affiliations:** 1 Department of Medical Cell BioPhysics, University of Twente, 7522 NB Enschede, The Netherlnds; p.liu-2@utwente.nl (P.L.); l.w.m.m.terstappen@utwente.nl (L.W.M.M.T.); 2 Department of Molecular Nanofabrication, University of Twente, 7522 NB Enschede, The Netherlands; p.jonkheijm@utwente.nl

**Keywords:** CTC, rare cell detection, immunomagnetic separation, magnetic nanoparticle, ferrofluids, non-specific binding, positive enrichment

## Abstract

**Simple Summary:**

For the enrichment of very rare cells, such as Circulating Tumor Cells (CTCs), immunomagnetic enrichment is frequently used. For this purpose, magnetic nanoparticles (MNPs) coated with specific antibodies directed against cancer cells are used. In this review, we look at the properties such a particle needs to have in order to be used successfully, and describe the different methods used in the production of such a particle as well as the methods for their separation. Additionally, an overview is given of the antibodies that could potentially be used for this purpose.

**Abstract:**

Here, we review the characteristics and synthesis of magnetic nanoparticles (MNPs) and place these in the context of their usage in the immunomagnetic enrichment of Circulating Tumor Cells (CTCs). The importance of the different characteristics is explained, the need for a very specific enrichment is emphasized and different (commercial) magnetic separation techniques are shown. As the specificity of an MNP is in a large part dependent on the antibody coated onto the particle, different strategies in the coupling of specific antibodies as well as an overview of the available antibodies is given.

## 1. Introduction

Magnetic separation has been used as early as 1792 [1], but was for a long time restricted to already magnetic materials. It is therefore not surprising that the first successful use of magnetic separation of blood cells was the separation of inherently paramagnetic red blood cells from different cell populations [2]. Usage of MNPs for cell separation was later demonstrated by Molday et al., who used polymeric microspheres to separate lymphocytes and red blood cells based on surface markers [3]. These first separations on abundant cell populations have initiated the development of multiple MNPs and separation techniques to perfect the magnetic separation of all types of cell populations, including rare cell types, such as Circulating Tumor Cells (CTCs). For the enrichment of CTCs, the most used method is positive enrichment via immunomagnetic separation. The only FDA-cleared system for the enumeration of CTCs is the CellSearch system, which uses MNPs for automatic enrichment of CTCs from a 7.5 mL blood sample [4].

When using MNPs to separate a specific rare cell population, it is important to consider the characteristics of both the MNPs and the magnetic configuration used. Over the years, many different particles have been proposed for CTC enrichment, and some have been used. Although there have been other reviews on the synthesis of MNPs [5,6,7,8,9], they generally focus on the synthesis process of the bare MNPs, and seldomly further explore their biological applications, especially the specific application of rare cell separation. As a consequence, the MNPs discussed are often not suitable for biological applications due to the toxicity of the chemical reagents used in the synthesis method. In this review, we will discuss the ideal characteristics of the MNPs for rare cell separation with a focus on CTCs and describe the existing techniques that can be used to make MNPs suitable for this purpose. Additionally, we will briefly discuss the magnetic configurations used and give an overview of the existing particles.

## 2. Immunomagnetic Particles for Rare Cell Enrichment

CTCs are extremely rare, especially in patients without known metastasis [10,11]. In these patients, the presence of one or more CTC in 7.5 mL of blood is significantly associated with a higher chance of disease recurrence and death [10,11]. With such a low frequency of CTCs, a considerable proportion of patients with one CTC in 7.5 mL of blood will be missed purely based on the statistical distribution of the CTCs [12]. Although the actual recovery of CTCs in immunomagnetic enrichment cannot be determined in the patient’s blood, as the actual number is not known, one can deduct that 30–90% of the CTCs are recovered from experiments in which tumor cells were spiked into blood [13]. This percentage varies based on the number of target antigens present on the CTC and the characteristics of the MNPs and the magnetic separation configuration. From these facts one can conclude that, to reliably detect the presence of CTCs in such low numbers, the blood volume will have to be significantly increased [14]. To examine the presence of CTCs in larger blood volumes, Diagnostic Leukapheresis (DLA) has been introduced [15,16], through which the mononuclear cell fraction, which also contains the majority of CTCs, can be obtained from the complete blood volume. Through this method, the number of CTCs, which were recovered from a DLA of 5 L of blood in metastatic patients, was dramatically increased. This increase is needed to be able to perform phenotypic and (epi-)genetic characterization for all patients, as well as secretome analysis, expansion, drug testing and introduction in animal models. Additionally, this increase in sample volume was used to improve the sensitivity of the CTC detection in order to reliably detect CTCs in non-metastatic patients [16]. Whereas, for a metastatic patient where only 1 CTC is found in a 10 mL blood draw, the number of CTCs found will go up to 500 CTCs upon increasing the volume to 5 L; the number of CTCs that is found in non-metastatic patient is much lower [10,11]. Whether the objective is to obtain a sufficient number of CTCs in order to determine the most effective treatment or whether it is to detect their presence in patients with no known metastasis, different requirements need to be met; as for the latter objective, it is important to have a process that allows for a comparison between patients, while for the former objective it is the minimum number of CTCs that is important.

**Figure 1:**
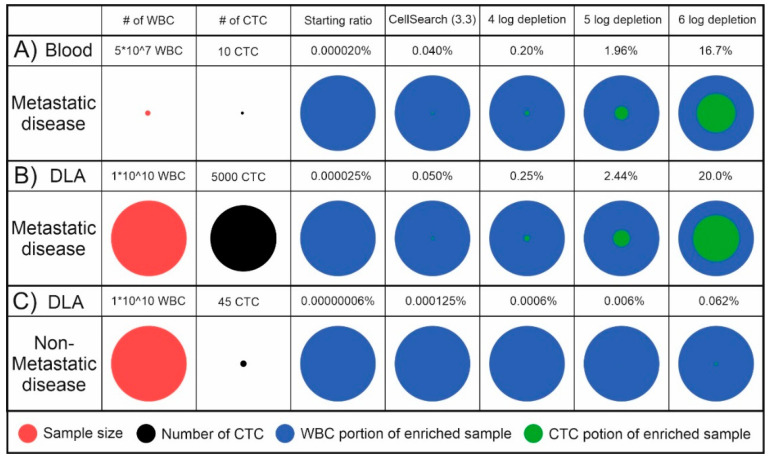
Theoretical comparison of the sample size, number of Circulating Tumor Cells (CTCs) and CTC and White Blood Cell (WBC) ratios in the starting material and enriched samples with increasing levels of WBC depletion. (**A**) 10 mL blood of a metastatic cancer patient containing 10 CTCs; (**B**) 100 mL DLA with 10^10^ mononuclear cells of the same metastatic cancer patient containing 5000 CTCs; (**C**) 100 mL DLA with 10^10^ mononuclear cells from a non-metastatic cancer patient with 45 CTCs that can lead to overt metastasis.

To get a feel for the number of cells that can be expected when processing DLA, we took as an example a blood sample from a metastatic cancer patient with 10 CTCs, a DLA of 5 L of blood from that same patient and a DLA of 5 L of blood from a non-metastatic patient. For the number of CTCs in the non-metastatic setting, we took as a reference the level of sensitivity needed to detect a tumor at the point when the 5-year risk of metastasis is 1%, which is 9 CTCs/L [17]. The effect of different levels of leukocyte depletion, assuming a CTC enrichment efficiency of 100%, is visualized in Figure 1. In practice, both the efficiency of the CTC collection during a DLA procedure as well as during enrichment will be less than 100%, making the CTC ratio even lower.

The background of white blood cells that remains after the enrichment is an obstacle for many (single cell) analysis techniques. In our experience, a background level of less than 10,000 leukocytes is needed for efficient single cell analysis. If it would be possible to perfect the immunofluorescent labelling of the enriched cell suspension and achieve a perfect detection of CTCs among leukocytes and other objects by fluorescence microscopy or flowcytometry, one could tolerate a higher background level. Fluorescently labelled antibodies can, however, non-specifically bind to non-target antigens through, for example, their Fc-receptors, and a background of 0.1–0.01% is quite common [18]. This non-specific binding increases when intracellular antigens are targeted as the fluorescently labelled antibodies are more prone to stick to the intracellular components. The use of multicolor antibody panels can significantly decrease impact of this background as usually the background affects all colors. Besides using at least one positive identifier of the target cell, one negative identifier already increases the specificity of the detection as well. The ability to detect a CTC among other cells, after immunofluorescent staining, depends also strongly on the number of antigens present on the target cells of the CTCs. This numbers of antigens can strongly vary and is a variable that cannot be controlled. A general rule for rare cell detection is therefore to use the fluorochrome with the highest intensity and smallest coefficient of variation after staining, as well as the lowest non-specific binding, to identify the target cells.

## 3. The Characteristics of an Ideal Magnetic Particle for Cell Separation

**Figure 2:**
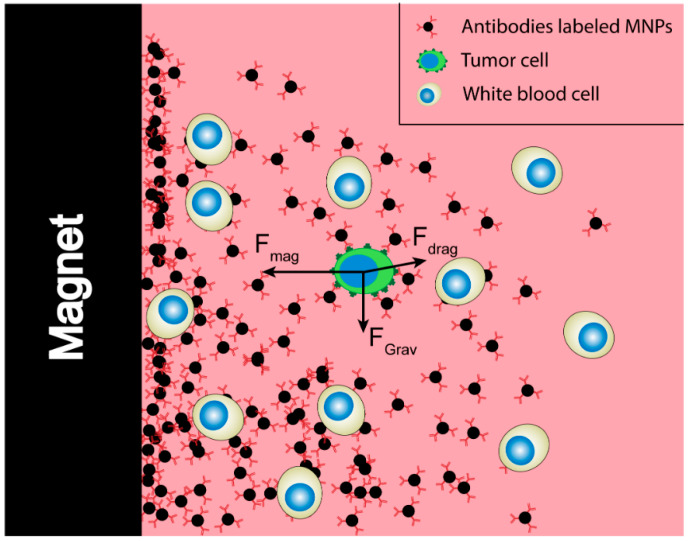
Virtual force analysis of the target cells during immunomagnetic separation.

Ideally, MNPs and the system for their separation allow for fast, selective separation of one or more subpopulations of cells. The first requirement of such a particle and the used separation setup is that the force exerted onto the cell through the bound MNPs is sufficient for it to be separated from the non-target cells. In order to assess the magnitude of this force in relation to the other forces at play, Chen et al. conducted a force analysis on the target tumor cells combined with MNPs [19]. Under the external magnetic field, the movement of target cells in the flow environment is affected by the magnetic force (*F*_mag_), the hydrodynamic drag force (*F*_drag_) and the net gravitational force after accounting for buoyancy. As the magnetic force (about 10^−10^ N) received by the cell is estimated to be much larger than the net gravitational force (about 10^−12^ N), only *F*_mag_ and *F*_drag_ are considered to simplify the calculation [20]. A scematical overview of the forces during a magnetic separation can be found in Figure 2. 

The force exerted by a single magnetic particle (*F_p_*) is the result of the magnetic moment (|M|) of the particle and the gradient of the magnetic flux density, ∇*B*, as seen in Equation (1):

(1) $$F_{p}=\left|M\right|\nabla B$$

This magnetic moment of the magnetic nanoparticle is given by Equation (2):

(2) $$\left|M\right|=\frac{V_{p}∆\chi_{p}}{\mu_{0}}B$$

Here, $\mu_{0}$ is the magnetic susceptibility of a vacuum, a constant, making the magnetic moment of a single magnetic particle dependent on

- the volume of a single magnetic nanoparticle (*V_p_*);
- the magnetic susceptibility of the magnetic nanoparticle (Δ*χ_p_*);
- the magnetic field generated by the external magnetic flux source (*B*).

As the cells are pulled through the surrounding liquid toward the magnet, they will experience a drag force. Assuming a laminar nature of the flow, and therefore a low Reynolds number, this force can be estimated using Stokes’ law. Here, the drag force is given as a function of the viscosity *η*, particle radius *r* and velocity *v*, as seen in Equation (3).

(3) $$F_{d}=6\pi \eta rv\;$$

The magnetic force on a cell is the sum of the forces of all the MNPs bound to the cell. As these forces will balance out, we can determine the resulting velocity using Equation (4), where N indicates the number of particles bound to the cell.

(4) $$v=\frac{N\;V_{p}∆\chi_{p}}{12\pi \eta r\mu_{0}}\nabla B^{2}$$

Based on this equation, as many MNPs as possible should attach onto the cell surface (*N*), and a magnetic configuration with a sufficiently large magnetic field (*B*) and the highest possible magnetic field gradient (∇*B*) needs to be used to separate the cells; the ideal magnetic nanoparticle should thus have a high magnetic susceptibility (Δ*χ_p_*) and a large volume (*V_p_*). Not considered in these equations is the ability of MNPs to bind to their target. With an increase of particle size, the particles will at some point become non-colloidal, causing them to sink to the bottom quickly, without having a chance to attach to the target. The limit at which a particle is no longer colloidal can range from around 10 nm to several micron [21]. This large range is due to its dependence not only on particle size and density, but also on the particles shape and surface chemistry, as these together with the suspending solution determine the particles surface charge [22]. This non-colloidal nature in combination with a decrease in stochastic movements [23] and the steric hinderance that effects larger particles will cause a decrease in binding efficiency. One avenue used to increase the possible collisions between target cells and nanoparticles is to expose the sample to changing magnetic gradients. In the CellTracks Autoprep, this is, for example, achieved by “magnetic incubation”, which involves moving the magnets to and from the sample tube multiple times during sample incubation as well as rotation of the sample tube [4].

Although, for an optimal capture, it would seem best to use particles for which very few or even a single bound particle will cause the target cell to be captured; the statistical probability of it being captured due to a non-specific interaction will increase when the number of non-specific interactions needed for capture decreases. 

### Superparamagnetic and Magnetic Susceptibility

The highest magnetic susceptibility is achieved by using ferromagnetic materials, in particular Fe_3_O_4_, also known as magnetite. The downside of this type of particle is that it remains magnetic when the external field is removed, resulting in nanoparticle clumping. This can be prevented by using paramagnetic materials, but these have a much lower susceptibility.

**Figure 3:**
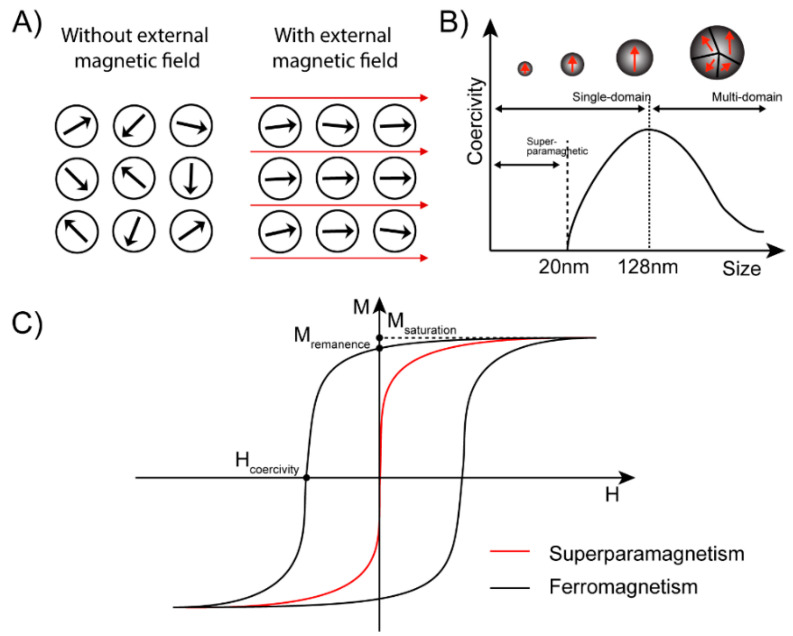
Overview of different properties of ferromagnetic and superparamagnetic materials (**A**) Schematic diagram of single magnetic domain direction with or without an external magnetic field. (**B**) The relationship between coercivity and size of Fe_3_O_4_; Reprinted with permission from [24]. (**C**) Hysteresis characteristic of the ferromagnetic and superparamagnetic nanoparticles (NPs).

The solution lies in superparamagnetic nanoparticles, which are ferromagnetic particles small enough to consist of a single magnetic domain. When an external magnetic field is applied, the magnetic domain inside the nanoparticles will be aligned instantly and have the same magnetic direction as the external magnetic field (Figure 3A). When the magnetic field is removed, this magnetic alignment remains, causing the particle to stay magnetic. How much alignment is retained is determined by the retentivity of the material. The strength of an opposing magnetic field needed to reduce this field to 0 is called the coercivity and is used as a measure of how magnetic a material has become. If, however, the size of a particle is below the critical value (~15–20 nm), the thermal energy will cause spontaneous magnetization reversal, resulting in the particles being randomly aligned with 0 coercivity (Figure 3B) [24]. In Figure 3C, it can be seen that by using superparamagnetic particles there is no remaining magnetization after removal of the magnetic field. In this way the agglomeration of nanoparticles in the absence of an external magnetic field can be avoided.

It is generally believed that the critical size of superparamagnetic nanoparticles is below 20 nm [24,25]. This means that their magnetic properties are also affected by the spin canting effect. This effect is caused by a lack of complete alignment in the spins of the surface atoms and leads to a lower magnetization of small-sized MNPs [26].

**Figure 4:**
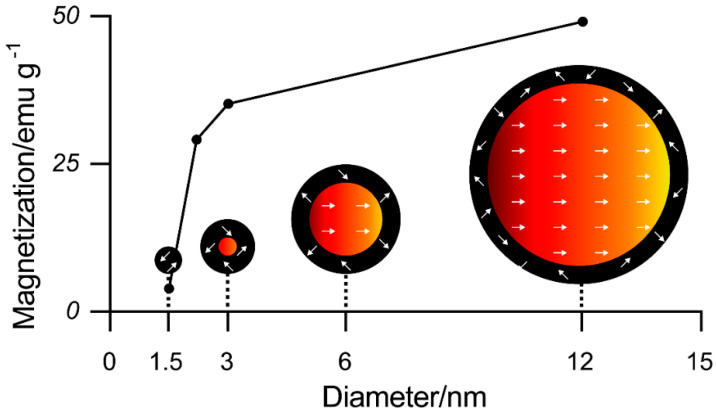
Magnetization value of the iron oxide nanoparticles with different sizes at 300 K and a visual description of the spin canting effect in various sizes of iron oxide nanoparticles (tilted layer = 0.9 nm). Red represents the magnetic cores and black the disordered shells. Reprinted with permission from [26].

Iron oxide nanoparticles can be considered as composed of a magnetic core and a magnetically disordered shell. The thickness of this spin-canted surface layer of the nanoparticles is 0.5–0.9 nm for all particle sizes [26]. This means that the relative portion of the spin-canted layer increases for smaller particles while the portion of the magnetic core and thereby the magnetization of the particles increases for larger sizes (see also Figure 4). This results in the ideal magnetic particle size being just below the superparamagnetic limit.

Daou et al. [27] studied two different coatings and reported that inorganic–organic interactions on the surface of nanoparticles can also affect the magnetic properties of nanoparticles. For example, the carboxylate coupling agent causes spin canting in the oxide layer, thereby reducing the saturation magnetization, but the phosphonate molecule does not.

As a superparamagnetic particle is at maximum around 20 nm, the particle volume is extremely limited and single superparamagnetic nanoparticles are not quickly attracted by magnets. This size limitation of the superparamagnetic particles can be circumvented by the agglomeration of many superparamagnetic nanoparticles together in a matrix of non-magnetic material in order to get both the wanted superparamagnetic behavior as well as an increased volume. Various methods have been used to prepare large-parameter superparamagnetic particles for biological separation, and the current commonly used sizes are from 50 nm to 4.5 μm.

**Figure 5:**
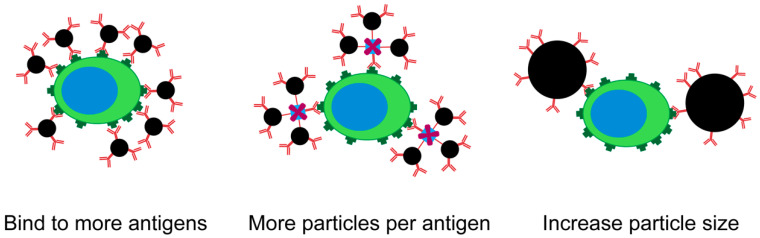
Schematic representation of the three ways to increase the amount of magnetic material coupled to a target cell.

Whereas in Equation (4) the number of MNPs was depicted as a single factor, McCloskey et al. proposed a formula to express the magnetophoretic mobility of immunomagnetically-labeled cells to reflect the number of particles bound. They split this number of bound particles up into three contributing factors, as can be seen in Figure 5 [28].

The elaboration of Equation (4) in this way leads to the velocity of a labeled cell being expressed by

(5) $$v=\frac{ABC\psi N_{a}V_{p}∆\chi_{p}}{12\pi \eta r\mu_{0}}\nabla B^{2}$$

Here, *ABC* is the antibody binding capacity of a cell population, *ψ* is the secondary antibody binding amplification factor and $N_{a}$ is the number of MNPs bound to a single antibody.

**Figure 6:**
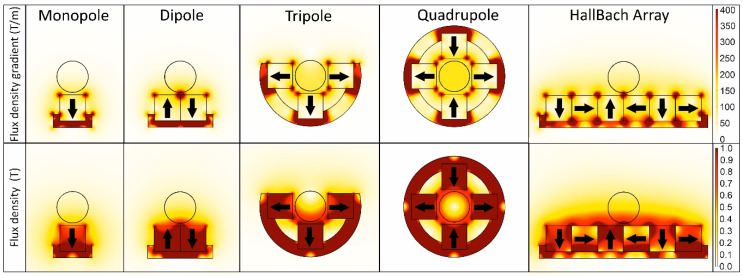
Magnetic flux and flux density for the same size magnets in different magnetic configurations. The position of a circular tube containing the sample is shown in each configuration.

These factors can increase the volume of magnetic material attached to the cell and thereby its magnetic moment. The force exerted on the cell is then determined by Equation (1), in which this magnetic moment all particles attached to the cells is multiplied by the magnetic field gradient. For this reason, the highest magnetic force is achieved when the magnetic field is sufficiently strong to reach the saturation magnetization of the particle used while creating the highest possible magnetic field gradient throughout the sample volume. Figure 6 shows the COMSOL 5.4 modelling of 10 mm by 10 mm N52 magnets in different yoked configurations with their magnetic flux density and flux density gradient.

Which configuration is best depends on the magnetic particle as well as on the size and shape of the container used. The dipole configuration is often used as it is simple and the cells and particles are collected at a single point. This configuration can be extended form an array of dipoles, essentially a row of magnets with alternating orientations. When using small magnets in this way, a high magnetic force can be achieved over a short distance. For cylindrical tubes, a quadrupole is often the most fitting configuration, and therefore it also was used for sample preparation in the original CellSearch system [29]. However, in order to be able to move the magnets away from the tube without the need for lateral movement, a tri-pole is used during the first steps in the commercialized CellSearch system [4]. In later steps, a dipole is used as it causes the collection of MNPs and cells at a single position in the tube, facilitating efficient resuspension without the need to vortex [4]. The Halbach array is more complex to assemble but has the benefit of the magnetic field reaching further away from the magnet compared to an array of dipoles.

Using larger magnets will increase the reach of the magnetic field, but at a loss of the magnetic gradient. By using very small magnetic elements, the magnetic gradient can be increased, resulting in a large force over a short distance. Osman et al., for instance, used micromagnets, resulting in a high gradient close to the magnets, but therefore needed the cells to be very close to the surface to allow capture [30]. Another way to create a high local field gradient is to introduce very small parts of ferro or (super-)paramagnetic material in close proximity to the cells. The material will be magnetized by the external magnetic field and become magnetic, creating very high magnetic gradients over a short distance, allowing the separation of cells in close proximity. This type of magnetic field gradient enhancement is employed, for instance, in Miltenyi separation columns as well as several microfluidic magnetic separation chips [31,32,33].

As can be seen in Equation (2), the magnetic field is also of influence on the magnetic moment of the particles. As depicted in Figure 3C, this relation between the applied magnetic field and the resulting magnetization and, therefore, the magnetic moment of the superparamagnetic particles, is not a linear relation but is limited by the saturation magnetization. When the saturation magnetization of the particles is reached, a further increase in the magnetic field will not increase the magnetic force. In this regard, it is important to realize that when the magnetic properties of particles are discussed, the saturation magnetization is often used as a parameter to express magnetic force, while perhaps with the configuration used, especially in the region furthest from the magnets, the field might be insufficient to reach this level of magnetization. The saturation magnetization is dependent on the synthesis method used to create the particles.

## 4. Synthesis Methods of MNPs

The synthesis of MNPs can be done via either physical or chemical methods. Physical methods usually require harsh reaction conditions, and the shape and particle size are difficult to control. Gas phase deposition and electron beam lithography are commonly used examples of physical methods. Chemical methods include co-precipitation, solvothermal reactions (hydrothermal reactions), thermal decomposition (hydrolysis and thermolysis of precursors), microemulsions, sol-gel synthesis and sono-chemical synthesis. For magnetic cell separation, almost exclusively particles synthesized via chemical methods have been used. Table 1 lists four synthesis methods to produce MNPs, including some of their characteristics. It also shows examples of TEM images of MNPs of different sizes and made through different processes. The merits and drawbacks of the four methods are described below.

### 4.1. Co-Precipitation

Co-precipitation is one of the most commonly used methods for preparing superparamagnetic iron oxide nanoparticles, which can be traced back to 1852 [42]. Almost all initial MNPs were created by co-precipitation. For the first cell separation, as was reported by Molday in 1977, polystyrene beads were used, but in 1982 Molday et al. first synthesized iron oxide particles coated with dextran by co-precipitation with ferric chloride, ferrous chloride and dextran [43]. Almost all MNPs developed in the following decades were created by co-precipitation. The diameter of these roughly spherical iron-dextran particles was 30–40 nm, including a 10–20 nm Fe_3_O_4_ core [43]. These ferrofluids are the precursor of the commercially available MACS^®^ MicroBeads made by Milteyni Biotec, which have a size of 50 nm.

The reaction process is to add alkali to an aqueous solution of a certain proportion of ferrous salt and ferric salt. Then, the reaction shown in Equation (6) occurs to obtain iron oxide nanoparticles.

Fe^2+^ + 2Fe^3+^ + 8OH^−^ → Fe_3_O_4_ + 4H_2_O (6)

The co-precipitation method has the advantages of being a low-cost, simple process that can be used on a large scale. However, the bare Fe_3_O_4_ nanoparticles agglomerate easily due to their extremely high surface energy and will bind non-specifically with biological components. In order to prevent this issue, a ligand, such as dextran, BSA or PEG, is commonly added during preparation. Alternatively, the surface of the particles is coated after preparation.

Ugelstad et al. first synthesized polystyrene particles of uniform size in 1976 [44]. The size could be adjusted between 0.5 and 20 μm, and then the particles were magnetized by the incorporation of particles made using the co-precipitation method. This process was patented in 1988; this is the basis of the commercially available Dynabeads.

By mixing soluble iron salts of Fe^3+^ and Fe^2+^ in a ratio of 1:2 together with latex, iron ions enter the polymer particles. The polymer particles contain functional groups, such as amino groups, to fix the iron ions. The last step is to raise the pH to form hydroxides, causing the Fe_3_O_4_ MNPs to precipitate the iron ions and become fixated inside. The obtained MNPs contain a uniform concentration of magnetic iron oxide, which is generally above 5%.

Owen and Liberti et al. first mixed the iron salt directly with a BSA solution, and then raised the pH value to generate Fe_3_O_4_ by co-precipitation in situ to prepare the magnetic particles [45]; later, the method was improved, and BSA was used to coat the already prepared Fe_3_O_4_ nanoparticles to obtain a type of MNP they called “ferrofluids”. The BSA coating reduces the non-specific binding and is rich in functional groups that can be used to bind streptavidin or antibodies. This process was patented and became the core technology for the fabrication of the MNPs used in the CellSearch system.

Feng et al. used PAA as a ligand and made hydrophilic magnetic nanoparticles through the co-precipitation method to enhance the potential encoding capacity of their beads called MRBLEs [46].

Although it serves as the basis of many commercial MNPs, the co-precipitation method also has some shortcomings. For example, the crystallinity of the product obtained by this method is low, which will affect its magnetic strength.

### 4.2. Thermal Decomposition

This method generally uses iron salt as a precursor, oleic acid as a ligand and octadecene as a solvent to prepare superparamagnetic iron oxide nanoparticles at a high temperature (e.g., 320 °C) [47]. The product obtained by the thermal decomposition method has the advantages of a controllable and narrowly distributed particle size and high magnetization, but at the same time, because the long-chain hydrocarbons used to control the shape and size also form the hydrophobic surface of the nanoparticles, surface modification is required to make them hydrophilic and biocompatible; therefore, some researchers have also tried to directly synthesize hydrophilic nanoparticles. [48].

Xu et al. [38] used this method to prepare oil-soluble nanoparticles of about 30 nm, and then coated them with amphiphilic polymers containing carboxyl groups to obtain nanoparticles with good biocompatibility and reactive carboxyl groups on the surface. The HER-2 antibody was then conjugated to the surface of the particle through covalent linkage of the amino and carboxyl groups.

Unni et al. [37] synthesized ferric oleate from acetylacetone and oleic acid, and then mixed it with octadecene and injected it into dodecane at a stable rate to obtain iron oxide nanoparticles with a diameter of 20.3 ± 1.5 nm. The surface was coated with streptavidin functionalized PEG to reduce the non-specific binding and allow the biotinylated anti-EpCAM (Epithelial Cell Adhesion Molecule) antibody to be bound to the surface of the nanoparticle.

Alternatively, Ge et al. [49] used PEG–(COOH)_2_ as a ligand to synthesize the hydrophilic magnetic nanoparticles in a one-step process through thermal decomposition. Here, the size of the MNPs can be adjusted between 4.2 and 17.5 nm by changing the length of the PEG and the ratio of the reactants.

### 4.3. Solvothermal

The solvothermal method is very similar to the hydrothermal method but uses a non-aqueous solvent. The method is carried out by sealing a reaction mixture in a Teflon-lined autoclave and then placing it at a temperature higher than the normal boiling point of the solvent. The solvent is then in a critical state, which greatly promotes the dissolution of the material. The method is easy to operate and can be prepared on a large scale. At present, researchers can already use this method to control the product morphology and particle size.

For example, Kim et al. [40] used this method to synthesize hydrophilic superparamagnetic nanoclusters with an adjustable particle size in the range of 100–300 nm in one step. The nanoclusters consisted of 11–13 nm nanocrystals. Then, by encapsulating the silica shell, a core-shell structure was obtained, which further improved its biocompatibility, and to which antibodies were attached to capture cells.

Zhang et al. [39] used ferrous sulfate as a precursor and PEI as a surfactant to synthesize iron oxide nanoclusters in a mixture of ethylene glycol (EG) and diethylene glycol (DEG) using a one-step solvothermal method. Then, the surface was modified with a leukocyte membrane to reduce the non-specific binding of the nanoparticles, and the aptamer SYL3C, specific for EpCAM-positive tumor cells, was bound on the surface.

### 4.4. Microemulsion

Microemulsion is a method to synthesize particles with a controlled shape and size. A binary system of microemulsion (water/surfactant or oil/surfactant) can be formed by different types of self-assembled structures (such as spherical and cylindrical micelles), so that the desired particle growth, nucleation and coalescence can be achieved [5].

Vidal-Vidal et al. [41] used the microemulsion method to encapsulate nanoparticles during their formation by co-precipitation in order to synthesize MNPs. The application of these particles in a biological application, however, requires extensive washing and stabilization treatments due to the remaining surfactants.

## 5. Methods to Bind Targeting Ligands on Magnetic Particles

In order to bind a magnetic particle to a specific cell type the surface of the particle can be coated with ligands that specifically bind to antigens present on the surface of the targeted cell type. Most frequently, antibodies recognizing the cell’s species-specific antigens are used for this purpose. The antibody–antigen immunological binding has a high specificity, which is needed to capture rare cells in the blood. The specificity and sensitivity of the immunoactive magnetic particle can be separated into the characteristics of the antibodies used and the characteristics of the magnetic particle. For the latter, the non-specific binding of the base particle can be influenced by the ligand-binding method, while for the antibody its affinity for its target and the density of expression on the target and non-target cells is of importance.

### 5.1. Antibodies and Ligands Used for CTC Enrichment

Antigens that are solely expressed on cancer cells have not been reported. One antigen that comes closest to this is the VAR2CSA malaria protein [50], which is not present on non-cancerous cells of hematopoietic origin and expressed on the cell surface of cancer cells but not their normal counterpart. An exception is the presence of the trophoblast on non-cancerous cells in the placenta. To identify CTCs in blood, one generally relies on antibodies that do not recognize antigens expressed on cells of hematopoietic origin or which are expressed at levels that are orders of magnitude lower as compared to the target cancer cells. In most cases monoclonal antibodies are used, as they are more specific as compared to polyclonal antibodies. As such, the monoclonal antibodies have less non-specific binding; additionally, they can be produced in a reliable and reproducible manner. Another option to capture a specific cell type consistently are aptamers. These chemically synthesized molecules are smaller in size and can be conjugated to nucleotides or functional groups. Aptamers have been increasingly used as targeting ligands for CTCs for detection. Due to the low cost and better stability of aptamers, they can be applied under a wide range of conditions [51].

The most commonly used antibody for CTC enrichment is the anti-EpCAM antibody. The EpCAM antigen is expressed on cells of epithelial origin and as such on most carcinomas [52,53]. As EpCAM is not expressed on blood cells, it can be used to capture CTCs from many different types of cancer, including lung, prostate, colon, breast and bladder cancer [29]. The most used system for the immunomagnetic enrichment of CTCs is the CellSearch system, which uses anti-EpCAM antibody-labeled ferrofluids to capture CTCs, and it has been shown that the EpCAM-positive CTC load is strongly correlated with a poor prognosis [54,55,56]. However, relying on EpCAM also has some limitations, such as its different expression levels between different subtypes of tumor cells; also, its expression level being downregulated during the epithelial–mesenchymal transformation (EMT) process may cause false negative results [52]. Due to the EMT, some tumor cells can change from a high to a low EpCAM expression and can no longer be captured using anti-EpCAM targeting ligands [57]. As EMT may promote the spread of tumor cells in the body, also these cells are of great interest to capture. Other antigens targeted to capture CTCs from the peripheral blood include Her-2 [58,59], EGFR [59,60,61,62], CD146 [13,63,64] and MUC-1 [65,66,67,68]. Additionally, the malaria rVAR2 protein and oncofetal chondroitin sulfate have been introduced as markers for CTC enrichment [69,70,71,72,73].

In Table 2, a list of antigens recognized by antibodies that are generally not expressed by blood cells is provided. The table is sorted by the presence of the antigens on epithelial cells followed by endothelial cells, neural cells and mesenchymal cells. Their expression on normal tissue and the various types of cancer can be found in the protein atlas (https://www.proteinatlas.org/search/). For the antigens where the (attempted) use for CTC capture is known, references are included. From the table, the large differences in expression on the cells of origin becomes clear. Each antigen may have different epitopes recognized by antibodies that do not cross react and their affinity for the target can be quite different, leading to differences in specificity and sensitivity for target antigen detection. For CTC enrichment, the importance of testing multiple antibodies directed against EpCAM and the possibility of an increase of capture by combining multiple low-affinity antibodies has been shown [74]. Detection efficiency for a single antigen could be increased by using different antibodies against the same antigen. Antigens expressed intracellularly, such as the cytokeratins, are more difficult to use for immunomagnetic enrichment, but not impossible, as shown by the use of small Milteny ferrofluids targeting intracellular cytokeratins [75]. Presence of the target solely in the nucleus makes this challenge even greater.

When using these different markers, different populations of tumor cells are captured [76]. In order to capture these populations at the same time, a combining of different markers could be used. In an attempt to use additional markers to increase the amount of CTCs captured, Beck et al. replaced part of the EpCAM particles in a CellSearch run with Her-2-, EGFR- and MUC-1-coated particles [88]. This change, however, decreased the capture efficiency. Wu et al. [89] compared EpCAM-based enrichment with a combination of EpCAM and MUC-1 in a similar fashion and also saw a decrease compared using to only EpCAM-coated particles. It seems the amount of EpCAM particles needs to remain sufficient for the capture of cells that are only or mostly EpCAM positive. One could increase the total amount of MNPs in order to keep the efficiency of the EpCAM capture; this will, however, also cause an increase in the number of healthy cells captured, thereby lowering the specificity of the test. It might be that different antibodies should be bound to the same magnetic nanoparticle in order to use multiple markers in order to capture CTCs more effectively.

Another way could be to use indirect labeling through biotin-labelled antibodies, which, in turn, can be captured using streptavidin-coupled MNPs. In this way the total amount of ferrofluid does not need to be increased to be able to use multiple antibodies at the same time. The downside of this approach is that the unbound antibody needs to be washed away before capture of the bound antibody can effectively take place.

### 5.2. Binding Antibodies to Magnetic Particles

There are several ways that can be used to bind antibodies to the surface of MNPs. The most commonly used method is via the streptavidin–biotin interaction, in which streptavidin-immobilized MNPs are coated with biotinylated antibodies through the high affinity interaction between streptavidin and biotin. The streptavidin–biotin interaction is one of the strongest non-covalent interactions in nature (K_d_~10^−14^ M). Its strength is considered to be approximately 10^3^ to 10^6^ times higher than an antigen–antibody interaction and remains stable under different pH environments [90,91,92]. The affinity of free biotin for avidin is even higher, but in almost all cases, streptavidin is used. Streptavidin and avidin have similar structures, both having four binding sites for biotin, but due to avidin being glycosylated it has a high isoelectric point (pI = 10), causing non-specific binding to become a problem when using it. As streptavidin is not glycosylated, it has a low isoelectric point (pI = 5–6) [93,94], and is widely used due to its lower non-specific binding.

Covalently binding streptavidin to the surface of MNPs through amino-carboxyl condensation is the most commonly used method, and antibodies can be indirectly attached to the MNPs through this method [37,95]. Li et al. [96] also successfully attached streptavidin to the surface of amino nanoparticles using glutaraldehyde as a linker. Similar to the principle of binding streptavidin on the surface of nanoparticles, it is possible to covalently bind antibodies directly to the surface of MNPs by condensing the amino carboxyl groups through EDC (1-Ethyl-3-(3-dimethylaminopropyl)carbodiimide)-NHS (N-hydroxysuccinimide) chemistry [37,95]. Li et al. [96] also successfully attached streptavidin to the surface of amino nanoparticles using glutaraldehyde as a linker. Similar to the principle of binding streptavidin on the surface of nanoparticles, it is possible to covalently bind antibodies directly to the surface of MNPs by condensing the amino carboxyl groups through EDC (1-Ethyl-3-(3-dimethylaminopropyl)carbodiimide)-NHS (N-hydroxysuccinimide) chemistry [38,40]. As the application of magnetic beads in scientific research has become common, magnetic beads bound with streptavidin or antibodies have been commercialized and can be easily bought.

## 6. Characterization of Magnetic Particle Performance

### Low Non-Specific Binding and No Agglomeration

When separating a cell type with a high frequency from blood, it is in most cases not very problematic if a small percentage of the cells in the collected fraction are of a different cell type. If, however, the frequency of the population of interest declines, the presence of these unwanted cells becomes more problematic as their relative percentage in the resulting sample increases.

**Figure 7:**
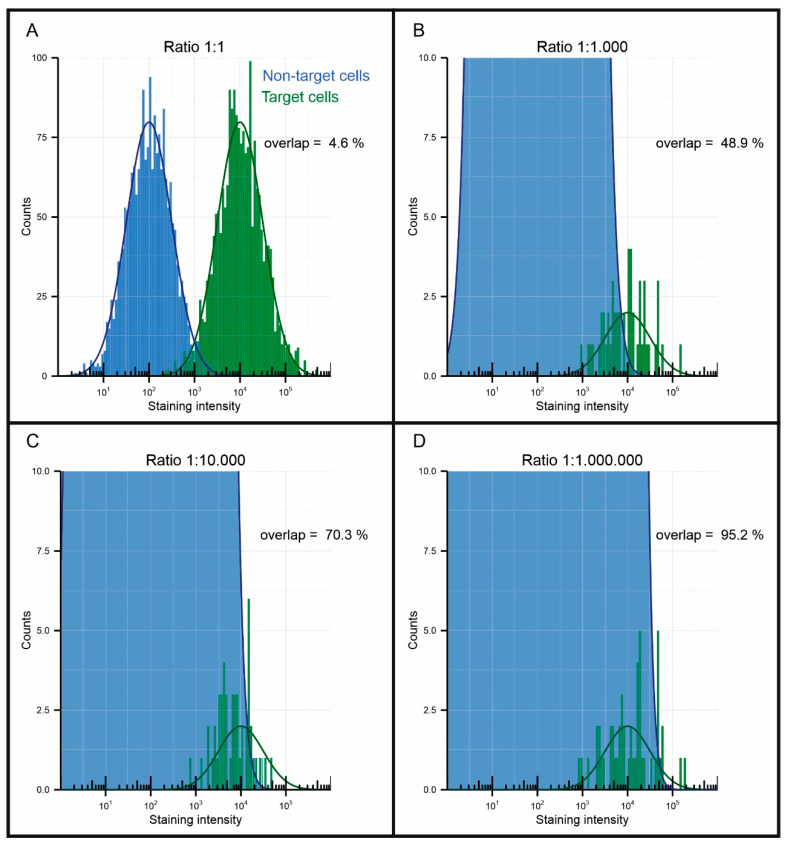
Visualization of the interference of non-target cells to the detection of target cells when using a target to non-target ratio of (**A**) 1:1, (**B**) 1:1,000, (**C**) 1:10,000 and (**D**) 1:1,000,000. Reprinted with permission from [98].

As an example, the EpCAM enrichment of the CellSearch system aimed at the enumeration of CTC has a 3.3-log depletion, but the average number of contaminating leukocytes in the resulting sample is still around 25,000 [97]. In contrast, the number of tumor cells is usually less than 10 [29]. Immunofluorescent labeling is used to discriminate the tumor cells from the leukocytes, but still some false positives are found in healthy individuals [29]. Andree et al. visualized the probability distributions of the staining intensity occurring on target and non-target cell populations, see figure 7 [98]. When the ratio of their numbers is 1:1, 1:1000, 1:10,000 and 1:1,000,000, respectively, 4.6%, 48.9%, 70.3% and 95.2% of the stained cells could not be distinguished. One way to resolve this overlap is to introduce multiple parameters to distinguish tumor cells from healthy cells. As can be seen from Table 2, many targets are available; however, they are often only expressed on a subset of tumor cells and many will be needed to distinguish the majority of the CTCs. Even if a marker expressed on all tumor cells can be found, for an enriched DLA sample with a ratio of 1:1,000,000, it has been estimated that five different parameters will be needed to distinguish the majority of the target population [99]. For this reason, it is of the utmost importance that a magnetic particle, in order to be useful for the separation of rare cell populations, does not, or only very rarely, bind to cells in a non-specific manner.

Non-specific capture of non-target cells can occur at different stages of the cell separation process. For example, cells binding to the nanoparticle surface, binding to a part of the antibody in a different manner (for example, Fc receptor binding) or cells getting trapped during separation. In some cases, it can also be that the target antigen is expressed in low amounts by a small fraction of the non-target cells, for instance the expression of MUC-1 in T-cells, leading to an increase in false positives [100].

Non-specific binding to the surface of nanoparticles is generally thought to be caused by physical absorption caused by electrostatic forces or hydrophobic interactions [101]. Furthermore, bare MNPs will agglomerate due to high surface energy and cause non-specific binding of other blood components due to the coordination reaction between the iron ions and the carboxyl groups in the protein, so the surface needs to be made up of molecules that do not show this type of interaction.

For this purpose, the particle surface is often coated to prevent the aggregation of the bare MNPs. Commonly used coatings are PEG [49], polyacrylic acid (PAA) [46,102], polyvinylpyrrolidone (PVP) [103], polydopamine (PDA) [104], dextran [105] and BSA [106,107]. The electrostatic repulsion or steric hindrance between these coated surfaces can prevent the agglomeration of nanoparticles, while at the same time non-specific binding is reduced due to the exposure of bare nanoparticles being avoided. Similarly, short-chain molecules such as PEG are also used as blocking agents to reduce non-specific adsorption. A clear overview of the different methods used for the surface modification of MNPs can be found in the review of Zhu et al. [108]. Besides the particle surface, other factors, such as the salt concentration and pH value of the solution, as well as the presence of a surfactant, can also affect non-specific binding.

There are also attempts to reduce the non-specific trapping of non-target cells during the separation process by designing unique structures. As these methods are not directly related to the magnetic nanoparticles used we will not go into details here [109].

## 7. Separation of Magnetic Particles in Systems for CTC Enumeration

Magnetic enrichment of cells was commercially introduced by Miltenyi in 1989 [33]. Today, the most used system for CTCs is still the CellSearch system, the only method cleared by the FDA for clinical CTC enumeration. The system enriches CTCs from 7.5 mL blood samples using EpCAM-functionalized ferrofluids for enrichment and fluorescent markers for labeling of the enriched sample. To date, clinical data involving more than 4700 patients has confirmed the value of CellSearch for predicting prognosis. The large size and high cost of the device limit the popularity of CellSearch system. Other commercial systems exist for the separation of magnetically bound CTCs, such as the Miltenyi *autoMACS* and *MultiMACS*, the Invitrogen *KingFisher* systems as well as magnetic separators for manual enrichment, such as BDs *IMag*, Invitrogens *Dynamag* or StemCells *Easysep*. Other methods have been developed, such as the *MagSweeper*, currently licensed to Illumina, in which a magnetic rod is moved through the solution in order to capture the magnetically labelled cells [110]. Most research into magnetic separation, however, is aimed at small, low-cost devices, and we will shortly describe these efforts here.

In order to capture the CTCs, MNP microfluidic chips are in these cases most often used in combination with external magnets [111]. In these chips, the capture efficiency is largely dependent on the magnetic array used. By using a micromagnet array that combines long-range permanent magnets and short-range thin-film micromagnets, it is possible to enhance the capture and control the distribution of the magnetic field up to the micrometer scale [39,112,113]. In order to capture the CTCs, MNP microfluidic chips are in these cases most often also used in microfluidic devices in combination with external magnets to capture the CTCs [111]. In these chips, the capture efficiency is largely depend on the magnetic array used. By using a micromagnet array device that combines long-range permanent magnets and short-range thin-film micromagnets, it is possible to enhance the capture by controlling and control the distribution of the magnetic field up to the micrometer scale [39,112,113].

When the surface of the microchannel is flat, the liquid will flow through in a laminar flow pattern. If the binding of MNPs to cells needs to take place within the chip, this laminar flow pattern causes insufficient contact between the MNPs and target cells. For this reason, Stroock et al. designed microstructures on the inner surface to produce lateral flow and effectively mix solutions [114]. Herringbone structured chips have also been designed in order to create turbulence and facilitate the capture of CTC on the chips surface [115,116,117]. In order to maintain the captured CTC trapped in the chip while still allowing easy recovery, dead-end side chambers can be used [109]. The combination of this approach with an increasing magnetic gradient facilitates the classification of CTC by their level of antigen expression [118]. The designed microstructures on the inner wall surface produce lateral flow in order to effectively mix solutions [114]. Herringbone structured chips have also been designed in order to create turbulence and facilitate the capture of CTCs on the chip’s surface [115,116,117]. In order to maintain the captured CTCs trapped in the chip while also still allowing easy recovery, dead-end side chambers can be used [109]. The combination of this approach with an increasing magnetic gradient facilitates the classification of the CTCs by their level of antigen expression [118].

By using a porous membrane impermeable to cells as a capture surface, the unbound particles can be separated from the captured cells while the possible volume flow might also increases [119]. When using a larger pore size but making the filter surface itself magnetic, the distance of the cells to the magnetic surface can be decreased to facilitate capture [120].

Instead of magnetically trapping the cells in the chip, it is also possible to divert them to a different outlet port to facilitate separation [121,122,123]. This can be done using a positive separation with superparamagnetic particles, but also a negative depletion using diamagnetic particles is possible [124,125]

Not only chip-based approaches have been used for the magnetic enrichment of CTCs; other methods have also been developed using magnetic rods wrapped in plastic [126] or using MNPs with positively charged surfaces [127].

As shown, DLA can be used to increase the sampling volume, but some researchers have also focused on capturing CTCs in vivo, directly from the patients’ blood, by using immunomagnetic enrichment. This can be done via an extracorporeal chip. Here, the blood is extracted from the patient, meaning labeling and separation takes place outside of the patient [128]. Another option involves a magnetic wire that can be placed intravascularly to separate the injected MNPs and CTCs directly from the blood stream [129].

These different efforts to extract CTCs are almost all reported with a proven level of recovery using one or more cell lines, sometimes even patient samples. When comparing these technologies on their benefit, it is, however, important to not only focus on the possible recovery, but also the usability of the resulting sample for downstream single cell isolation and analysis. In many cases, a low sample purity, the inability to extract all of the captured cells or the used fixation will impede the success of the downstream single-cell isolation technologies, as well as the (epi-)genetic, protein or functionality assay. There are many different single-cell isolation and analysis technologies available, which have different sample requirements. An overview of the existing single-cell isolation technologies can be found in the review of Valihrach [130], while other reviews focus on the different types of analysis possible after isolation [131,132,133].

## 8. Conclusions

For the enrichment of CTCs using MNPs, the characteristics of the particles are of great importance. We have discussed the different characteristics of MNPs and their impact on the enrichment of CTCs. We emphasized the importance of the high-purity enrichment for CTC identification and isolation, especially for large sample volumes. Common synthesis and surface modification methods as well as different strategies for the use of immunomagnetic particles are discussed with a focus on improving specificity. Panels of multicolor antibodies that recognize specific targets with low non-specificity increases the reliability of CTC detection, hence we have listed the available antigens for the readers’ reference. The characteristics of the different magnetic configurations were compared and finally we briefly introduced some of the existing applications of immunomagnetic particles for tumor cell enrichment.

In practice, insufficient depletion caused by the non-specific binding of MNPs to white blood cells hinders the identification of CTCs and the reduction of these cells can greatly facilitate their identification. In recent years, great progress has been made in the synthesis of MNPs in the field of materials chemistry and it would be very beneficial if this progress could be implemented into cell separation applications. In addition, although people often use blocking reagents to reduce non-specific binding, the extent and cause are rarely reported. Due to the rarity of CTCs, larger sample volumes will need to be processed, potentially in vivo, making the specificity of the binding even more important.

## Figures and Tables

**Table 1:** Different synthetic methods and the characteristics of the products obtained.

Method	Size	Morphology	Magnetization (Emu/g)	Ligand	Surface Groups Used	Supplier	Photos	Reference
Co-precipitation	30–1000 nm	Roughly spherical	-	Dextran	−OH	Miltenyi biotec (MACS)	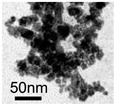 (A)	[34,35]
	100 nm	Irregular shape	-	BSA	−NH_2_−COOH	Veridex Ferrofluid™	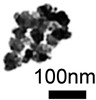 (B)	[29]
	1.0/2.8/4.5 μm	Spherical	10.8–23.5	Polymer	−NH_2_	Invitrogen^TM^ Dynal	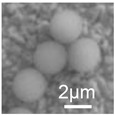 (C)	[36]
Thermal decomposition	20.3 ± 1.5 nm	Spherical	85.8	PEG	−COOH	N/A	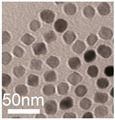 (D)	[37]
	30 nm	Spherical	-	ABC triblock copolymer	−COOH	N/A	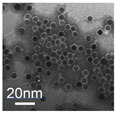 (E)	[38]
Solvothermal	80 nm	Spherical nanocluster	80	leukocyte membrane	Hydro-phobic inter-action	N/A	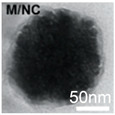 (F)	[39]
	100–300 nm	Spherical nanocluster	58.48	Silica	−OH	AMO LIFESCIENCE Inc., Korea	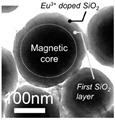 (G)	[40]
Microemulsion	10 nm	Spherical	33.2	Oleyla-mine	−NH_2_	N/A	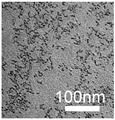 (H)	[41]

Picture (C) reprinted with permission from [36]; Picture (D) reprinted with permission from [37]; Picture (E) reprinted with permission from [38]; Picture (F) reprinted with permission from [39]; Picture (G) reprinted with permission from [40]; Picture (H) reprinted with permission from [41].

**Table 2:** Overview of the antigens (used) for immunomagnetic tumor cell enrichment.

CD #/Abbr.	Name	Expression	Location	Used for CTC Capture
Epithelial cells and no or few hematopoietic cells				
CD44		ep	cs, ic	Yes [76]
CD49f	Integrin Alpha 6	ep	cs	Yes [76]
CD66e	CEA (CAM5)	ep (colon)	cs	No
CD66f	PSG1	ep, pl, li	cs	No
CD104	Integrin beta4	ep, ed, ne, ke	cs	No
CD118	LIFR	ep	cs	No
CD133	Prominin-1	ep, hs, ed, ne	cs	No
CD164 (1)	MUC-24	ep (prostate cancer, bone mets)	cs	No
CD164 (2)		ep (bowl, lung, thyroid)	cs	No
CD164 (3)		hs, sc (bone marrow)	cs	No
CD175	Tn	ep, sc	cs	No
CD175s	Sialyl Tn	ep, ed, erythroblasts,	cs	Yes [77]
CD176	Thomsen–Friedenreich antigen	ep, sc	cs	No
CD227	MUC-1	ep, sc	cs	Yes [65,66,67,68]
CD283	TLR3.	ep, fi, ne, dc	cs, ic	No
CDw293	BMPR-1B	ep, ms, bc	cs	No
CD318	CDCP1	ep, hs	cs	No
CD325	N-Cadherin	ep, ne, mu (cardiac), fi, pa, li	cs, ic	Yes [78,79,80]
CD326	EpCAM	ep, sc (embryonic)	cs	Yes [29,54,55]
CD331	FGR1	ep, ed, fi, ms, mu (cardio), ne	cs	No
CD331-4	FGFR1-4	ne, ki, te		No
CD332	FGFR2	ep, li, fi, ne,	cs, ic	No
CD340	erbB-2 kinase/ Her-2	ep (breast), ed, ke, hs, sc	cs, ic	Yes [58,59]
CD344	Frizzled-4	ep, ms, ne, ki, te	cs	No
CD338	ABCG2	ep, li, ki, lu, pl	cs	No
CD350	Frizzled-10	ep (colon), ki, lu, br, pl	cs	No
CK	Cytokeratins 1-23	ep	ic	Yes [75]
AR	Androgen receptor	ep	ic, in	No
EGFR	Epithelial Growth Factor/Her-1	ep, pl, pa, ki, li	cs	Yes [59,60,61,62]
ER	Estrogen receptor	ep	ic, in	No
PSMA	Prostate-Specific Membrane Antigen	ep (prostate)	cs, ic	Yes [81,82,83]
Trop2	Tumor-associated Ca signal transducer 2	ep	cs	Yes [76]
Endothelial cells and no or few hematopoietic cells				
CD62E	E-selectin	ed (activated)	cs	Yes [84,85,86,87]
CD144	VE Cadherin	ed, sc	cs	No
CD146	MCAM	ed, sm, me, dc, T-cells (activated)	cs	Yes [13,63]
CD201	EPCR	ed	cs	No
CD202b	TIE2	ed, angioblasts	cs	No
CD228	Melanotransferrin	ed, sc, me	cs	No
CD248	Endosialin	ed (tumor), fi	cs	No
CD266	TWEAK R	ed	cs	No
CD299	C-type lectin domain family 4 member M	ed, li, lymph node	cs	No
CD300g	CLM-9	ed	cs, ic	No
CD309	VEGFR-2	ed, sc	cs	No
CD322	JAM-B	ed (high)	cs	No
CD362	Syndecan-2,	ed, sm (vascular), fi, me	cs	No
Neural cells and no or few hematopoietic cells				
CD246	ALK	ne (embryonic CNS & PNS	cs, ic, in	No
CD271	NGF receptor	ne, ms (bone marrow)	cs	No
Mesenchymal cells & no or few hematopoietic cells				
CD90	Thy-1	fi	cs	No
CD167b	DDR2	bc, he, lu	cs, ic	No
CD292	BMPR-1A	mu (skeletal), bc, ms	cs	No
CD331-4	FGFR1-4	ne, ki, te	cs	No
CD339	Jagged1,	st (bone marrow), ep (thymic), ed, ne, ke, ov, pr, pa, pl, he	cs	No
CD333	FGFR	ne, ki, te	cs	No
c-Met/HGF	Hepatocyte growth factor	pl	cs	No
Vimentin	Vimentin	ms	ic	No
FAP	Fibroblast activation protein, Seprase	ep, ms, fi	cs	No
No or few hematopoietic cells				
VAR2	Malaria-encoded VAR2CSA	pl	cs	[69,70,71,72,73]

Ep = epithelial cells; ed = endothelial cells; hs = hematopoietic stem cells; ms = mesenchymal cells; pl = placenta; li = liver/hepatocytes; da = dendritic cells; ke = keranocytes; sc = stem cells; ki = kidney; pa = pancreas; lu = lung; fi = fibroblast; st = stromal cells; bc = bone cells; sm = smooth muscle; me = melanocytes/melanoma; te = testis; he = heart; ov = ovarian; pr = prostate; br = brain.
